# Influence of Ink Composition and Drying Technique on the Performance and Stability of Fe–N–C‐Based High‐Temperature Proton Exchange Membrane Fuel Cells

**DOI:** 10.1002/cssc.202500905

**Published:** 2025-07-14

**Authors:** Tanja Zierdt, Md Raziun Bin Mamtaz, Tom Eek, Julia Müller‐Hülstede, Steffen Rehse, Quentin Meyer, Dana Schonvogel, Peter Wagner, Chuan Zhao, Michael Wark, K. Andreas Friedrich

**Affiliations:** ^1^ Institute of Engineering Thermodynamics German Aerospace Center (DLR) Carl‐von‐Ossietzky‐Str. 15 26129 Oldenburg Germany; ^2^ Institute for Building Energetics Thermotechnology and Energy Storage (IGTE) University of Stuttgart Pfaffenwaldring 31 70569 Stuttgart Germany; ^3^ School of Chemistry The University of New South Wales NSW Sydney 2052 Australia; ^4^ Institute of Chemistry Carl von Ossietzky University Oldenburg Carl‐von‐Ossietzky‐Str. 9‐11 26129 Oldenburg Germany; ^5^ Institute of Engineering Thermodynamics German Aerospace Center (DLR) Pfaffenwaldring 38‐40 70569 Stuttgart Germany

**Keywords:** catalyst layer fabrication, electrocatalyst, Fe–N–C, freeze‐drying, high‐temperature proton exchange membrane fuel cells

## Abstract

Fe–N–C catalysts have emerged as a potentially cost‐effective alternative to Pt‐based catalysts in high‐temperature polymer electrolyte membrane fuel cell cathodes. However, the optimal design and deposition method of the Pt‐free catalyst layer remain unclear. Herein, the effect of conventional oven drying compared with freeze‐drying on the performance of commercial Fe–N–C catalyst layers is investigated. The gas diffusion electrodes are fabricated by doctor blade coating. Freezing the wet catalyst layer at –26 °C and subsequent sublimation of the solvents leads to a 45 % increase in mass‐normalized peak power density compared to the conventional oven drying. This is attributed to a templating mechanism of the solvents, resulting in a thicker catalyst layer and improved acid retention, which enables optimal reactant transport. In contrast, freeze‐drying with liquid nitrogen negatively impacts the catalyst morphology, leading to reduced porosity and performance. During 100 h of operation, the performance decreases by a similar magnitude, regardless of the fabrication method used. Operando electrochemical impedance spectroscopy with the distribution of relaxation times shows no catalyst deactivation through the fabrication methods. The results highlight the importance of optimizing catalyst layer fabrication methods for Fe–N–C catalysts to achieve improved performance in fuel cell applications.

## Graphical abstract

Fe–N–Cs are a promising alternative to Pt‐based catalysts in high‐temperature polymer electrolyte membrane fuel cells; however, their catalyst layer design requires optimization. This study compares conventional oven drying and freeze‐drying for catalyst layer fabrication using the same catalyst. Different impacts on the morphology are revealed. Freeze‐drying at −26 °C significantly increases the energy output of the fuel cell.

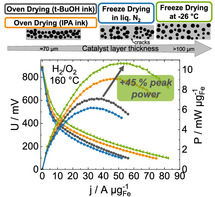

## 1 Introduction

The high‐temperature polymer electrolyte membrane fuel cell (HT‐PEMFC) operates at around 160 °C. This technology benefits from air cooling, easier heat and water management, reduced peripheral components, and weight savings, making them ideal for aviation compared to low‐temperature (LT)‐PEMFCs.^[^ ^1^, ^2^ ^]^ Additionally, the superior increased CO tolerance enables the direct use of reformate from renewable methanol and liquefied natural gas and further process gases, for example, in maritime settings.^[^ ^3^ ^]^ HT‐PEMFC electrodes require high Pt loadings of up to 1 mg_Pt_ cm^−2^ per electrode due to partial poisoning of Pt surface from phosphate ions from the phosphoric acid‐doped polybenzimidazole membrane.^[^ ^4^ ^]^ Pt is expensive and scarce and accounts for a large part of around one‐third of the total costs of PEMFC stacks and might become a bottleneck to meeting their cost targets for the upcoming decades.^[^ ^5^ ^]^ As a result, Pt‐free catalysts such as Fe–N–Cs are considered a promising alternative as they show comparable oxygen reduction reaction activity to Pt/C in acidic electrolyte, and benefit from a higher resistance to phosphate ion poisoning.^[^ ^1^, ^4^, ^6^, ^7^ ^]^

The substantially different properties of Fe–N–C compared to Pt‐based catalysts, such as acidophilic nature,^[^ ^8^ ^]^ lower volumetric activity resulting in thicker catalyst layer compared to Pt‐based electrodes,^[^ ^4^ ^]^ and active site location inside smaller meso‐ and micropores^[^ ^9^ ^]^ requires new strategies to optimize the catalyst layer fabrication methods and to harness the intrinsic activity of the catalyst on PEMFC level. An effective catalyst layer for HT‐PEMFC should generate a high amount of triple‐phase boundaries by optimal distribution of the phosphoric acid and reactant gases through its porous structure while maintaining its mechanical stability.^[^ ^1^ ^]^

We found in our previous study that the performance of Fe–N–C‐based catalyst layers for HT‐PEMFC can be influenced by its deposition method.^[^ ^4^ ^]^ Furthermore, we found that the performance of Fe–N–C in HT‐PEMFCs can be enhanced through optimizing the wetting properties by increasing the polytetrafluorethylene (PTFE) content of the catalyst layer to 20–50 wt%.^[^ ^10^ ^]^ The utilization of additives (surfactants), typically beneficial for Pt‐based gas diffusion electrode (GDE),^[^ ^11^ ^]^ was found to have a negative influence on the performance of the Fe–N–C‐based ones.^[^ ^12^ ^]^ Talukdar et al. revealed that freeze‐drying (FD) of Pt‐based electrodes for LT‐PEMFC improved performances, due to the resulting porosity from a thicker catalyst layer improving mass transport.^[^ ^13^ ^]^ This higher porosity was achieved during the partial sublimation of the solvent. Cyclohexanol was used as a solvent, as its ambient temperature melting properties make it more attractive and easier to freeze‐dry than isopropanol (IPA). FD is a sufficient tool to avoid particle agglomeration and achieve high porosity by templating a defined structure through the solvent, and it is used in various fields, such as aerogels,^[^ ^14^ ^]^ Nafion membranes,^[^ ^15^ ^]^ and other porous structures.^[^ ^16^ ^]^ However, FD has not yet been investigated for Fe–N–C catalyst layer fabrication for HT‐PEMFCs. Indeed, most HT‐PEMFC studies typically use isopropanol for Fe–N–C suspension with PTFE as a binder for GDE fabrication and spray coating.^[^ ^4^, ^17^, ^18^, ^19^, ^20^ ^]^ Heating of the substrate (gas diffusion layer (GDL) with microporous layer (MPL)) to 40 °C^[^ ^4^, ^10^, ^19^ ^]^ or 80 °C^[^ ^18^ ^]^ while spray coating the catalyst ink enhances the solvents (IPA and water) evaporation. Last, doctor blade coating is also used often to deposit the catalyst layer, with the freshly formed GDE placed in a low‐temperature oven (40 °C)^[^ ^4^ ^]^ to evaporate the solvent. Conventional drying methods (e.g., thermal drying, solvent evaporation) can cause densification (low porosity) due to the collapse of the pores, causing severe mass transport limitations.^[^ ^13^ ^]^

In our study, the impact of FD of Fe–N–C catalyst layers is investigated. Four GDEs are fabricated using doctor blade coating: 1) IPA‐based ink and oven drying, 2) *tert*‐butanol (*t*‐BuOH)‐based ink and oven drying, 3) *t*‐BuOH‐based ink and FD at −26 °C, and 4) *t*‐BuOH‐based ink and FD in liquid nitrogen followed by (partial) solvent sublimation. A quality control of the GDEs to ensure comparable catalyst loading and distribution and to identify wettability and layer thickness is done and reveals higher thickness for the freeze‐dried electrodes. The effect of the different drying methods of Fe–N–C‐based cathodes on the overall HT‐PEMFC performance is identified, including distribution relaxation times (DRT) analysis of the electrochemical impedance spectroscopy (EIS). Depending on the freezing temperature, both highly positive and negative impacts on the HT‐PEMFC performance are found. Moreover, the durability is tested for all cells for a duration of up to 120 h at a constant load of 100 mA cm^−2^.

## 2 Results and Discussion

**Figure 1:**
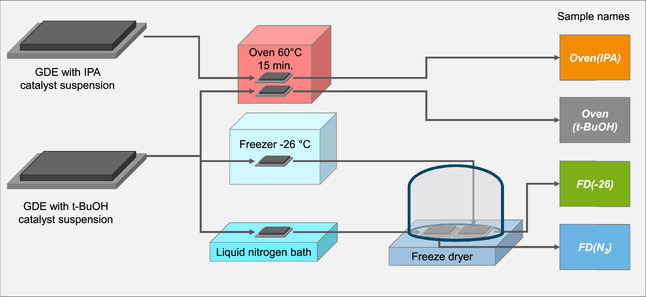
Schematic GDE fabrication and sample naming.

Four gas diffusion electrodes are fabricated, one using IPA for the Fe–N–C catalyst suspension and three with *t*‐BuOH as solvent and using different drying methods (**Figure** **1**). The drying procedure includes the typical oven drying for 15 min to evaporate the solvent to fabricate the GDEs denoted as Oven(IPA) and Oven(*t*‐BuOH). FD is examined as an alternative drying method. The impact of freezing the freshly coated GDE at −26 °C, denoted as FD(−26), and shock frosting in liquid nitrogen FD(N_2_) is investigated. Following the freezing of the catalyst layer, the GDEs were placed in a freeze‐dryer to sublimate the solvent.

*t*‐BuOH is chosen as a solvent for FD as IPA has a low melting point of −88 °C,^[^ ^21^ ^]^ which requires a greater effort in freezing, and a larger temperature decrease is needed for solidification of the catalyst layer. In comparison, *t*‐BuOH has similar physical properties to IPA, but a melting point of 26 °C^[^ ^22^ ^]^ and is therefore easier to handle for this process. Alternating the freezing temperature to −80 °C did not achieve proper catalyst layer homogeneity with the *t*‐BuOH suspension. Moreover, cyclohexanol was found to be an insufficient solvent for FD with this type of catalyst suspension. Detailed information can be found in the supporting information on page 2, accompanied by Figure S1, Supporting Information.

### 2.1 Physicochemical Properties of the Gas Diffusion Electrodes

The iron content of the GDEs is analyzed by inductively coupled plasma mass spectrometry (ICP‐MS). The Fe–N–C loading of the GDE is calculated by taking the Fe–N–C catalyst iron content of 0.47 ± 0.02 wt% into account (**Table** **1**).^[^ ^23^ ^]^ Except for Oven(*t*‐BuOH), which exhibits a higher loading, the iron contents are comparable for all in the range of 11.4–12.1 μg cm^−2^. An additional Oven(*t*‐BuOH) 2.0 was fabricated with a lower iron loading of 9.3 mg_Fe_ cm^−2^ (1.6 mg_Fe–N–C_ cm^−2^) and characterized to evaluate the effect of the loading.

**Table 1:** Iron and Fe–N–C catalyst loading.

Gas diffusion electrode	Iron loading [μg_Fe_ cm^−2^]	Fe–N–C loading [mg_Fe–N–C_ cm^−2^]
Oven(IPA)	11.4	2.5
Oven(*t*‐BuOH)	15.6	3.2
FD(–26)	11.4	2.3
FD(N_2_)	12.1	2.7

**Figure 2:**
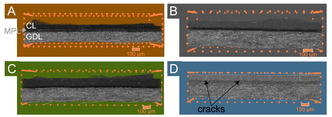
A–D) 3D μ‐CT images of the GDEs: A) Oven(IPA) with the position of the catalyst layer (CL), MPL, and GDL illustrated exemplarily; B) Oven(*t*‐BuOH); C) FD(–26), and D) FD(N_2_).

Iron elemental mapping with micro X‐ray fluorescence (*μ*‐XRF) reveals that all gas diffusion electrodes have homogenous iron distribution over the 25 cm^−2^ area (Figure S2, Supporting Information). X‐ray computed tomography (*μ*‐CT) (**Figure** **2**) of the GDEs shows a homogeneous catalyst layer structure, except the FD(N_2_) GDE has visible cracks within the catalyst layer (Figure 2D, S3D, Supporting Information). These cracks likely stem from the mechanical stress during the liquid nitrogen‐induced shock frosting.

**Figure 3:**
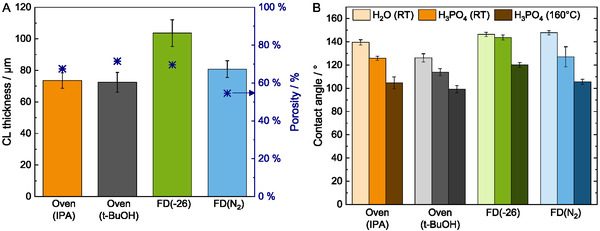
A) Results from evaluation of the μ‐CT images: catalyst layer thickness and catalyst layer porosity of the GDEs. B) Contact angles from the catalyst layer surface of the GDEs with H_2_O and conc. H_3_PO_4_ at room temperature and with conc. H_3_PO_4_ at 160 °C.

Analysis of the *μ*‐CT images of the Oven(IPA) and Oven(*t*‐BuOH) GDEs reveals similar catalyst layer thickness (73 ± 6 and 74 ± 5 μm) and porosities (68% and 72%) in **Figure** **3**A. The porosity values are calculated by determining the proportion of the structure to the void from the CT images. Therefore, changing the solvent seems to have a minor impact on these catalyst layer properties. Among the four GDEs, FD at ‐26 °C leads to the thickest catalyst layer (104 ± 9 μm), due to the templating mechanism by the solvent crystals, and is similar to the findings of Talukdar et al.^[^ ^13^ ^]^ Its 70% porosity is similar to the Oven(IPA) and Oven(*t*‐BuOH) catalyst layers. The FD(N_2_) GDE has a comparable catalyst layer thickness of 81 ± 5 μm to the Oven(IPA) and Oven(*t*‐BuOH) ones, but a lower porosity of 55%. The lower porosity might indicate a partially collapsed porous structure or lower initial pore formation, as the rapid shock freezing does not allow for the growth of large solvent crystals, unlike the FD(−26).

The surface wettability of the differently fabricated gas diffusion electrodes is investigated using contact angle analysis (Figure 3B) for water and H_3_PO_4_ at ambient temperature and 160 °C. Oven(IPA) has a water contact angle of 140 ± 2°. The catalyst layer surface of the FD(−26) and FD(N_2_) has a similar water contact angle at room temperature above 145°. Oven(*t*‐BuOH) has the lowest contact angle of 126 ± 4°. For Fe–N–C‐based electrodes, a high hydrophilicity is beneficial since the GDE is less prone to flooding, which is followed by a performance drop, as we discovered in our previous study.^[^ ^10^ ^]^

The contact angle of the catalyst layer with H_3_PO_4_ at room temperature for Oven(IPA) (126 ± 1°) is similar to FD(N_2_) (127 ± 9°). Oven(*t*‐BuOH) has the lowest acid retention with a contact angle of 114 ± 2°. The lower contact angles of Oven(*t*‐BuOH) originate from the higher loading, as the gas diffusion electrodes with similar loading have similar wettability. Oven(*t*‐BuOH) 2.0 (lower catalyst loading) has a higher contact angle of 138 ± 2° (H_2_O) and 136 ± 1° (H_3_PO_4_). During oven drying of Oven(*t*‐BuOH) (with higher loading), some PTFE might have migrated closer to the MPL,^[^ ^4^ ^]^ leaving the catalyst layer surface slightly less hydrophobic. The FD(−26) catalyst layer has the highest H_3_PO_4_ contact angle of 144 ± 6°. Interestingly, the FD(−26) GDE has a similar wetting behavior with H_3_PO_4_ as with H_2_O at room temperature. For the other GDEs, the contact angle with conc. H_3_PO_4_ is lower than with H_2_O.

For all GDEs, a reduction of the contact angle is observed at 160 °C compared to room temperature as the viscosity of H_3_PO_4_ decreases at higher temperatures.^[^ ^24^ ^]^ For Oven(*t*‐BuOH), the contact angle decreases by 13% and by 17% for the other ones. While Oven(IPA) has a higher acid retention than Oven(*t*‐BuOH) at room temperature, this effect is less pronounced at 160 °C. FD(−26) has the highest acid retention, which might stem from the thicker catalyst layer and/or a more homogenous PTFE distribution during the slower freezing process.

### 2.2 HT‐PEMFC Performances

**Figure 4:**
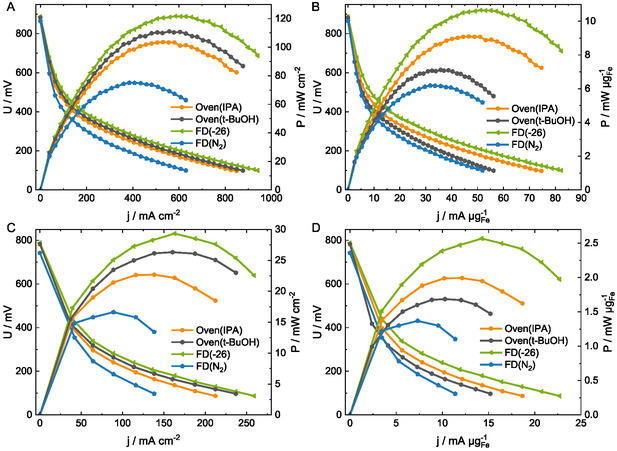
Results from the HT‐PEMFC single‐cell test at 160 °C. A) Area‐based polarization curves during H_2_/O_2_ (anode/cathode) operation, B) corresponding iron mass‐normalized polarization curves, C) area‐based polarization curves under H_2_/air operation, and D) corresponding mass‐normalized polarization curves.

The Fe–N–C‐based GDEs are combined with a commercial Pt‐based anode and phosphoric acid‐doped polybenzimidazole (PBI) membrane to form the membrane electrode assembly (MEA). The performance is evaluated in HT‐PEMFC at 160 °C under dry oxygen and air supply at the cathode, and dry hydrogen at the anode. The polarization curves recorded under H_2_/O_2_ (**Figure** **4**‐A) have similar open circuit voltages of 0.88 V for FD(−26), Oven(IPA), and Oven(*t*‐BuOH), and 0.86 V for FD(N_2_). The slightly lower open circuit voltage of FD(N_2_) MEA could indicate slightly less accessible active sites at the beginning of test (BoT) due to its lower porosity. The activation losses (<50 mA cm^−2^) are comparable, which is as anticipated, given that the same catalyst is used for all samples. In the ohmic predominant region (>50 mA cm^−2^), Oven(*t*‐BuOH), and FD(−26) MEAs have the lowest overpotential and therefore higher performance than Oven(IPA) and the FD(N_2_) ones.

The highest area‐based performance is observed for FD(−26), followed by Oven(*t*‐BuOH), Oven(IPA), and FD(N_2_) (**Table** **2** and S1, Supporting Information). The second MEA measurement performed for each GDE type verifies high reproducibility, except for FD(N_2_), signifying the inconsistency in the shock frosting process (Figure S4A–D, Supporting Information). An additional comparison to a commercial Pt‐based MEA (Celtec) is given in (Figure S5, Supporting Information), revealing the expected lower performance for commercial Fe–N–C, as also found in literature.^[^ ^6^, ^18^ ^]^

**Table 2:** HT‐PEMFC performance at beginning of test.

MEA	Peak power density H_2_/O_2_		Peak power density H_2_/air	
	[mW cm^−2^]	[mW μg_Fe_]	[mW cm^−2^]	[mW μg_Fe_]
Oven(IPA)	104	9.1	23	2.0
Oven(*t*‐BuOH)	116	7.4	27	1.5
FD(−26)	122	10.7	29	2.6
FD(N_2_)	75	6.2	17	1.4

To eliminate any impact of the catalyst layer loadings (Table 1), the current density is normalized to the iron content and plotted in Figure 4B. Oven(IPA) has a higher mass‐normalized performance (Table 2) than Oven(*t*‐BuOH), followed by FD(−26) which has the highest performance. The higher loading of *t*‐BuOH causes its lower mass‐normalized performance, with its performance only marginally higher than FD(N_2_). To eliminate the combined impact of FD and loading, the performance of the low loading “Oven(*t*‐BuOH) ‐2.0” was evaluated as well and displays an 8% lower mass‐normalized peak power density than FD(−26) (Figure S6, Supporting Information). This confirms superior performance (Table 2) of FD(−26) irrespective of the loading, indicating that the performance enhancement is due to FD process. In this instance, the templating mechanism of the solvents created a pore network, enhancing the accessibility of active Fe–N_*x*_ sites and facilitating optimal reactant transport.

**Figure 5:**
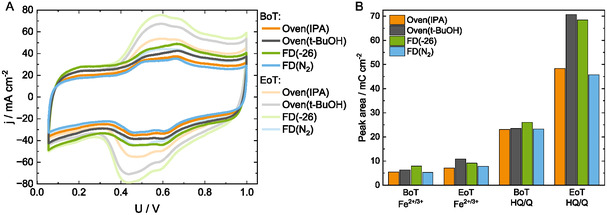
A) Cyclic voltammograms at BoT versus EoT with a scan rate of 100 mV s^−1^ during H_2_ (anode)/N_2_ (cathode) operation. B) Peak areas from the peak deconvolution and computations for Fe^2+/3+^ in the range of 0.7–0.9 V and for the HQ/Q peak in the range of 0.4–0.6 V.

Differences in the performance can be attributed to a slightly lower contact angle of *t*‐BuOH, thus more wetted with phosphoric acid, in line with the highest performance of FD(−26) and its highest acid retention. Moreover, FD(−26) exhibits the highest wetted carbon structure, as visible within the cyclic voltammograms (Figure S7A, Supporting Information, and **Figure** **5A**, discussed later in more detail), representing more accessible surface area, which is beneficial for accessing active Fe–N_*x*_ sites. Also, the higher porosity and thickness of this catalyst layer might increase catalyst accessibility and acid distribution and thereby positively impact the performance. Talukdar et al. also found improved performance for a Pt‐based freeze‐dried catalyst‐coated membrane in LT‐PEMFC tests. They attributed this to improved catalyst layer morphology due to higher porosity, reducing water flooding of the pores and charge transfer losses, and enhancing oxygen diffusion.^[^ ^13^ ^]^

In contrast, freezing with liquid nitrogen lowers the porosity, which might induce inferior reactant distribution, leading to lower performance. Furthermore, the cracks observed in the catalyst layer (Figure 2D and S3D, Supporting Information) likely led to the increased accumulation of phosphoric acid instead of a uniform distribution and could hinder oxygen transport to the active sites, thereby altogether reducing the performance. Additionally, FD(N_2_) has a lower reproducibility during the HT‐PEMFC operations (Figure S4D, Supporting Information), potentially due to a less controllable freezing process. Thus, shock freezing negatively impacts the homogeneity of the GDE and the HT‐PEMFC's performance as a result.

While maintaining the performance trend, the mass‐specific performance decreases by 76–80% using H_2_/air operation than H_2_/O_2_ for all MEAs (Figure 4), due to the lower oxygen saturation, as also observed by Gokhale et al.^[^ ^20^ ^]^

Overall, freezing the *t*‐BuOH catalyst layer at −26 °C and subsequent (partial) solvent sublimation positively influence the performance, while freezing the GDE in liquid nitrogen is detrimental.

### 2.3 Stability over 100 h

**Figure 6:**
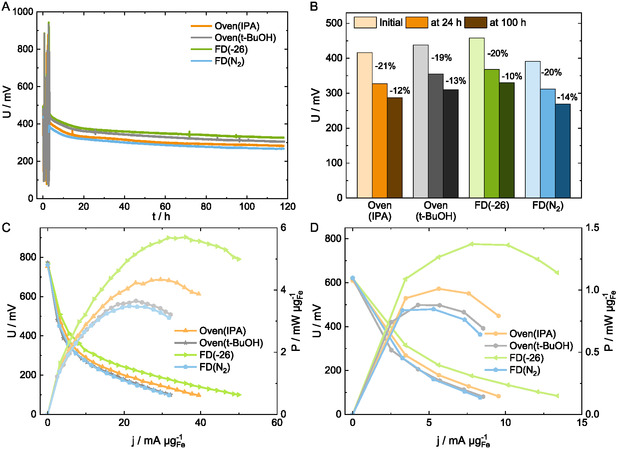
A) Voltage over time during H_2_/O_2_ stability at 100 mA cm^−2^. The spikes during the first hours belong to the BoT characterization. B) Voltages at 24 and 100 h of operation with indicated percentage losses in between. C) Mass‐normalized polarization curves at EoT during H_2_/O_2_ operation. D) Mass‐normalized polarization curves at EoT during H_2_/air operation.

The stability is evaluated at 100 mA cm^−2^ for at least 100 h (**Figure** **6A**). Similar voltage losses are observed for all HT‐PEMFCs irrespective of the cathode drying method (Figure 6B). The typically known initial loss is 20% after 24 h of operation for all MEAs, indicating similar degradation behavior, again consistent with the same catalyst used. Active site deactivation and demetallation account for this phenomenon in HT‐PEMFCs.^[^ ^6^, ^19^ ^]^ Afterward, the voltage loss is less intense, with a further 10–14% loss over the next 100 h, with FD(−26) being slightly more stable (10%). The higher acid retention and thicker catalyst layer might be assumed to be beneficial for the stability of FD(−26), as the electrode might be less prone to flooding with electrolyte and allowing a more sufficient active Fe–N_*x*_ site accessibility in comparison to the other MEAs. However, the voltage loss is highly similar for all MEAs. The degradation rates for all samples are closely similar (3.5 ± 0.3 mV h^−1^ within the first 24 h and 1.3 ± 0.1 mV h^−1^ within 24–100 h). These similarities are attributed to the same Fe–N–C catalyst used, thus indicating that the drying method has negligible influence on the stability of the catalyst.

After the stability test, the highest performance remains for FD(−26), with 46% loss in peak power density under H_2_/O_2_ and 47% under H_2_/air (Figure 6C,D, Table S1, Supporting Information). Oven(IPA) has the second‐highest performance, with a peak power density loss of 52% and 49% in H_2_/O_2_ and H_2_/air. The Oven(*t*‐BuOH) and FD(N_2_) have the same performance at the end of test (EoT) characterization, despite different decay rates and overall lower losses for the FD(N_2_). The lower porosity of the FD(N_2_) catalyst layer might be less intruded by phosphoric acid (excluding the cracks) and hindered iron site degradation, while the cracks possibly accumulated phosphoric acid. Contrastingly, the slightly higher loading of Oven(*t*‐BuOH) might affect the performance at EoT, for example, active site accessibility and deactivation as well as acid distribution. However, the differences are small, and the reproducibility of FD(N_2_) MEA is rather low.

The size and evolution of the redox peaks are analyzed using cyclic voltammetry (Figure 5A and S7, Supporting Information) in H_2_/N_2_, with peaks from 0.4 to 0.9 V belonging to the overlapping redox peaks of Fe^2+^/Fe^3+^ and hydroquinone/quinone (HQ/Q) species^[^ ^4^, ^10^, ^19^ ^]^ FD(−26) has the highest capacitive current densities due to its higher porosity and thicker catalyst layer, which comes along with larger electrochemical accessible catalyst surface area and more optimal wetting with the phosphoric acid, leading to the best performance. For peak analysis and separation, a peak fitting was applied (Figure S8 and S9, Supporting Information) to determine the respective charges (peak area) of the Fe^2+^/Fe^3+^ and HQ/Q redox peak (Figure 5B). The Fe^2+^/Fe^3+^ charge remains similar after the stability test, while the HQ/Q charges increase at EoT. This indicates that surface oxidation occurs to an extent and increases the HQ/Q peak at EoT, indicating carbon corrosion by reactive oxygen species, and is consistent with our previous observations.^[^ ^19^ ^]^

After HT‐PEMFC operation, the *μ*‐CT images of MEAs (Figure S10, Supporting Information) show retained structural integrity and no severe degradation of the oven‐dried and freeze‐dried catalyst layers over time.

### 2.4 Electrode Resistances and Oxygen Reduction Reaction (ORR) Rates at BoT and EoT

**Figure 7:**
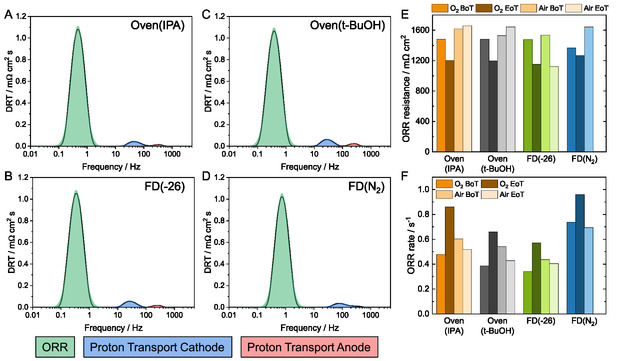
A–D) DRT plots from impedance spectra at BoT at 100 mA cm^−2^ under H_2_/O_2_ operation with peak assignment. E) ORR resistances determined from the peak area from BoT and EoT during H_2_/air operation. F) corresponding ORR rates at BoT and EoT.

DRT analysis is performed from EIS at 100 mA cm^−2^ (Figure S11, Supporting Information) for the oxygen and air operation for all MEAs at BoT and EoT. Three peaks are visible within the DRT plot in the low‐, intermediate‐, and high‐frequency regions (**Figure** **7**A‐D). These peaks can be assigned to ORR, cathode, and anode proton transport resistance, respectively.^[^ ^4^, ^25^ ^]^

ORR resistances at BoT are in the same range for all MEAs, with the exception of FD(N_2_) with a slightly lower value (Figure 7E). The ORR resistances are highly comparable to the values of a doctor blade coated Fe–N–C GDE with a similar commercial Fe–N–C (precious‐metal‐free (PMF)‐011904) from our previous study.^[^ ^4^ ^]^ The ORR resistances during H_2_/air operation are slightly higher but of a similar magnitude (Figure S12 and Table S2, Supporting Information). The comparable ORR resistances indicate that the different drying methods do not negatively impact the catalyst and that the performance differences (Figure 4) can be related to the catalyst layer structure. The ORR rates are also comparable except for FD(N_2_) MEA, which has a slightly higher ORR rate. However, this slightly higher rate and the lower resistance are obviously not translated into the performance since it is the lowest for FD(N_2_) (Figure 4). EIS was recorded at a fixed current density of 100 mA cm^−2^. At this current density, FD(N_2_) had a lower potential compared to the other MEAs, which could lead to slightly higher ORR rates. This was also observed in literature, where, for example, an increased overpotential leads to a higher ORR rate.^[^ ^26^ ^]^

The proton transport resistances are similar for all MEAs (Table S2, Supporting Information). It could be assumed that the higher thickness of the FD(−26), and the lower porosity of FD(N_2_) cathodes can influence the proton transport resistances, but this is apparently not the case, and further, the ORR resistance is predominant (highest peak area). Similarly, the anode proton transport resistances are similar for all samples, which indicates that the different cathode processes equally impact phosphoric acid diffusion to the anode (Table S2, Supporting Information).

For additional evaluation of the stability, the EIS data at EoT are also evaluated by DRT. The ORR resistance during H_2_/O_2_ operation is lower, and the rates are higher at EoT compared to BoT (Figure 7E). As surface oxidation is visible in the EoT voltammograms (Figure 5), some previously inaccessible active sites, which also contribute to the faster ORR rate, might become accessible. Moreover, the redistribution or accumulation of the phosphoric acid leads to higher accessibility of active sites and reduction of the resistance.^[^ ^19^ ^]^ However, this effect observed in DRT data is overshadowed by the catalyst degradation, leading to drastic performance losses after 100 h of operation (Figure 6).

During H_2_/air operation, the ORR resistances remain almost the same at EoT compared to BoT for Oven(IPA) and Oven(*t*‐BuOH), while they drop for FD(−26) (Figure 7E). The FD(−26) has a slightly lower ORR resistance and the highest performance in H_2_/air (Figure 4 and 6), where less oxygen is present and could lead to mass transport issues. This fabrication method is providing access to more active sites due to better porosity. Overall, a similar ORR rate is present (Figure 7F). It is obvious that the performance decreases (Figure 6), likely due to the known stability issues, such as carbon corrosion, demetallation, and loss of active catalyst sites of the Fe–N–C and HT‐PEMFC^[^ ^19^ ^]^ while the remaining catalyst sites are still active. For FD(N_2_), the DRT data from EoT during H_2_/air operation are not available, because no EoT EIS at 100 mA cm^−2^ could be recorded as the voltage was below the minimal voltage limit of the test protocol. The cathode and anode proton transport resistances reveal no unexpected behavior (Table S2, Supporting Information). To conclude, DRT proves that the catalyst itself was not inactivated or otherwise damaged due to the catalyst layer fabrication, with similar features observed to previous work.^[^ ^19^ ^]^

## 3 Conclusion and Outlook

This study highlights the impact of solvent choice and drying methods during cathode catalyst layer fabrication on the performance of Pt‐free HT‐PEMFC GDEs. Our findings confirm that FD is feasible for Fe–N–C‐based catalyst layer fabrication. FD of GDE at −26 °C leads to an up to **45% increase** in mass‐normalized peak power density compared to an MEA based on oven‐dried *t*‐BuOH ‐GDE‐ and **18% higher** than an MEA containing an oven‐dried IPA GDE. This performance boost is attributed to a positive templating effect during freezing, leading to a more optimal reactant transport and enhanced accessibility of the active Fe–N_*x*_ sites, resulting in a thicker catalyst layer and higher acid retention. In contrast, liquid nitrogen freezing resulted in lower porosity, lower performance, and reduced reproducibility. Furthermore, DRT analysis confirms that catalyst integrity remains intact across all catalyst layer fabrication methods, confirming that observed performance variations stem primarily from structural differences induced by catalyst layer processing techniques. The drying method does not have a negative impact on the stability since the average loss after 100 h is not more than 2 mV h^−1^ for all MEAs. The −26 °C freeze‐dried electrode indicates slightly more stable behavior.

Our research shows the improvement of Pt‐free HT electrodes by adjustment of the drying method during catalyst layer fabrication, which is important for the transfer of new Pt‐free catalyst with promising activity in rotating‐ring‐disc electrode experiments^[^ ^23^, ^27^ ^]^ to real HT‐PEMFC. Additionally, FD presents a potential pathway for solvent recycling, enhancing the sustainability of electrode fabrication and thus paving the way to a more circular economy.

## 4 Experimental Section

4.1

4.1.1

##### Determination of Catalyst Loading by Inductively Coupled Plasma Mass Spectrometry

To examine the iron content in the GDEs, inductively coupled plasma mass spectrometry (ICP‐MS) was conducted with the iCapQ device (Thermo Fisher Scientific). Alongside the 15 mg PMF, for each Fe–N–C GDE a 1 cm^2^ piece was punched out. About 2 mL HNO_3_ (RotipuranSupra 69 wt%, Carl Roth) was added, the solution was ultrasonicated for 5 min, and afterward boiled for 1 h at around 100 °C, while the level of 2 mL concentrated HNO_3_ was kept. The solution was stored overnight. Then, the sample volume was adjusted to 50 mL by the addition of ultrapure water and filtered. The calibration solutions consisted of a Fe ICP standard (Carl Roth) with concentrations of 0, 1, 10, 50, 75, 100, 200, and 300 μg_Fe_ L^−1^. Lutetium and germanium served as internal standards to monitor any drift in signal sensitivity. A correlation coefficient of at least 0.999 was ensured during calibration. The Fe–N–C loading was calculated by correlating the iron content of the catalyst powder to the determined iron content of the 25 cm^−2^ catalyst layer individually for each ICP‐MS measurement to vanish any measurement inaccuracy from the device (having a standard deviation of 0.02 wt% for the PMF throughout four measurements).

##### μ‐CT, 2D‐, and 3D Image Construction for Calculation of Structural Parameters

Pieces with a diameter of 6 mm of the GDE and MEA (in the center of the MEA) were punched out and vertically placed in the SkyScan 2214 Nano‐CT (Bruker). **Table** **3** shows the experimental parameters during measurement. The reconstruction was performed with NRecon software (Bruker), and a volume of interest (VOI) was extracted using the software DataViewer (Bruker) to generate 2D images of the GDE. For determining the catalyst layer thickness, the whole GDE thickness at 10 positions was determined at two representative cross sections using DataViewer software (Bruker) first. Afterward, the GDL/MPL width, determined in the same way from Freudenberg H23C2 GDL/MPL without the presence of a catalyst layer, was subtracted from the GDE thickness to finally receive the catalyst layer thickness. 3D images were received using the software CT_Vox_ (Bruker). The porosity was determined in the CT_An_ software (Bruker) from binarized images by calculating the proportion of the structure to the void of the total VOI. The size of the voxels was given by the resolution of the *μ*‐CT measurement (1.72 μm pxl^−1^), so that only the large macropores will be captured by this analysis, and the smaller pores are not considered.

**Table 3:** Experimental parameters of μ‐CT measurements.

Parameter	Value
Acceleration voltage [kV]	60
Current [μA]	100
Rotation step [°]	0.2
Random movement	4
Averaging frames	10
Resolution [μm pxl^−1^]	1.72
Exposure time [ms]	709
Stage temperature [°C]	Room temperature

##### Contact Angle

Contact angle measurements were carried out with the device OCA25 (Dataphysics), equipped with a temperature chamber TPC 160U (Dataphysics). Drops (9 μL) of ultrapure water (Millipore, Merck) or conc. phosphoric acid (85 vol% H_3_PO_4_ Emsure Merck) were deposited via a blunt cannula onto the catalyst layer of a horizontally aligned GDE. For water a cannula of 0.26 mm and for conc. phosphoric acid of 0.60 mm inner diameter were used. Analysis of contact angles was done using Young‐Laplace fitting within dpiMAX Software (Dataphysics). The H_2_O and H_3_PO_4_ contact angles of four independent measurement points were averaged for each electrode.

##### XRF

The elemental mappings were recorded with the X‐ray XGT‐9000 (Horiba Scientific, Germany) device with a rhodium tube with a tube voltage up to 30 kV. The analysis was performed under a helium gas atmosphere to increase sensitivity. Elemental analysis was achieved by means of the analysis of signals from the energy versus intensity graph, with the experimental setting according to **Table** **4**. Other elements like fluorine showed too low energy to interpret the results. Regular energy and intensity calibration were performed with Al‐ and Cu‐maintenance samples for the XGT‐9000 to ensure reliable data collection.

**Table 4:** Experimental parameters of XRF measurements.

Parameter	Value
Pixel size [pix]	128
Pixel time [ms]	30
Capillary diameter [mm]	1.2
Deadtime correction [‐‐]	20
He flowrate [mL min^−1^]	400
Current [μA]	Automatic
Voltage (max.) [kV]	30

##### GDE Fabrication

Commercial Fe–N–C catalyst (PMF‐014401, Pajarito Powder) was used for GDE fabrication. 240 mg catalyst powder was mixed with 1) 1600 mg isopropanol (99.5% for synthesis, Roth) and 2) 1440 mg *t*‐BuOH (99.5% extra pure, Thermoscientific) and 222 mg ultrapure H_2_O (Millipore, Merck). Water was added at this point to reduce the melting point of *t*‐BuOH and achieve a homogenous suspension. The suspensions were mixed on a roller mixer RS‐TR‐10 (Phoenix Instrument) at 45 rpm for the IPA ink and the *t*‐BuOH ink within a mixer mill (MM 400, Retsch) with a frequency of 5.5 Hz overnight (16 h) to ensure blending and wetting of the solvent with the catalyst powder. Afterward, the suspensions were sonicated in an ice‐cooled US bath for 15 min, followed by the addition of 1) 266.2 and 2) 246 mg PTFE‐dispersion (60 wt% in H_2_O, Sigma‐Aldrich). Followed by 3 h of US bath and afterward addition of the solvents for 1): 624 mg IPA and for 2): 562 mg *t*‐BuOH. The 1) IPA ink had a PTFE/catalyst ratio of 0.68, solid (catalyst + PTFE)/liquid ratio of 17.2, and solvent/catalyst ratio of 5.8, and the 2) *t*‐BuOH ink of 0.62, 16.7, and 6.0, respectively. The (2) *t*‐BuOH ink has slightly lower solid content to keep the ink viscosity similar, as the *t*‐BuOH is more viscous^[^ ^28^ ^]^ than IPA.^[^ ^29^ ^]^ The suspensions were further sonicated for 2.5 h and afterward applied to an area of 5 × 5 cm with a doctor blade (ZAA 2300, Zehntner) with a speed of 30 mm s^−1^. A GDL/MPL layer (H23C2, Freudenberg) served as substrate. The drying to remove the solvent of the catalyst layer varied for the four samples: 1) With the (1) IPA suspension: a) Oven(IPA): Drying at 60 °C at ambient pressure in an oven (VDL 115 Binder GmbH) with a fan speed of 30 rpm and ambient pressure. 2) With the (2) *t*‐BuOH suspension (same ink formulation for each GDE): a) Oven(*t*‐BuOH): Analog to “Oven(IPA).” b) FD(−26): The GDE (catalyst layer still wet) was placed into freezer with a temperature of –26 °C overnight (at least 16 h) until the complete solidified catalyst layer. The GDE was directly placed into a FD setup onto a precooled (−80 °C) aluminum block. The pressure was reduced to 0.54 mbar and held for 3 h to (partial) sublimate the solvent and allow the setup to get to room temperature. c) FD(N_2_): GDE (with still wet catalyst layer) placed on top of the liquid nitrogen bath (–196 °C). The GDL/catalyst layer was secured within a frame to prevent bending of the GDL due to the rapid change in temperature. Afterward, the FD procedure was analogously performed to “FD(−26),” but the aluminum block was precooled in liquid nitrogen.

For all GDEs, the mentioned step was followed by drying at 100 °C in an oven to remove the possible remaining solvent and also to keep the oven fabrication procedure similar to the FD method. All dry catalyst layers contained ≈40 wt% PTFE as binder. The GDEs were stored under a nitrogen atmosphere until use. The GDEs were cut for the HT‐PEMFC single‐cell test and the physicochemical analysis according to (Figure S13, Supporting Information).

##### MEA Assembling

A commercial H_3_PO_4_‐doped PBI membrane (55 ± 4 wt% acid concentration and 34 ± 3 mg cm^−2^ acid content, acc. to the supplier,^[^ ^30^ ^]^ Celtec P, BASF) was stored in 50 wt% H_3_PO_4_ (Merck) overnight to ensure consistent acid content. The self‐prepared cathode was placed on an aluminum plate and sealed with Kapton foil to fix the active area to 4.05 cm^−2^ (Trigona Fuel Cell Components GmbH). The membrane was positioned between the two foil layers, followed by the Celtec‐based Pt/C anode (Celtec High‐Temperature PEM Fuel Cell, P1100W, Fuel Cell Store) with a catalyst loading of ≈1 mg_Pt_ cm^−2^ (given by manufacturer). For hot‐pressing, 10‐mm‐thick aluminum shim shields (≈80% of the initial MEA thickness) were used to prevent MEA from too high mechanical stress. The MEA was hot pressed at 140 °C for 30 s under 1 kN force in a hot press (TRG 2, P/O/Weber), then thermally annealed at 160 °C for 30 min in air in an oven (VDL 115 Binder GmbH) to remove excess water. Thickness and weight were measured three times and averaged before analysis.

##### HT‐PEMFC Operation

**Figure 8:**
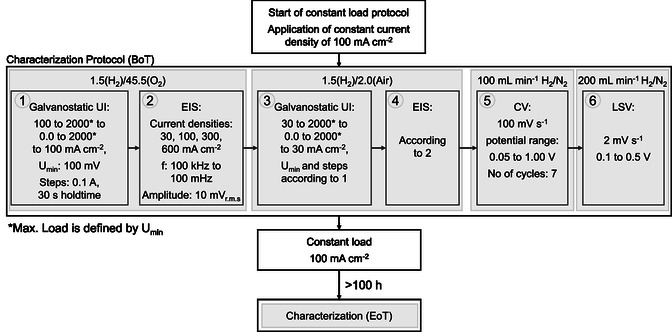
MEA characterization protocol for HT‐PEMFC single cell.

The MEA was fixed in a cell fixture (cF5/100 HT Gr V1.4) with serpentine graphitic flow fields from balticFuelCells. HT‐PEMFC tests were conducted on a commercial test station with software (Schubert GmbH) with a quickCONNECT fixture (qCF 5/100 HT, balticFuelCells). A cell compression of 0.75 MPa (477 mbar) was applied over the whole test duration. A leak test was carried out by applying nitrogen to both cathode and anode sides to fix a backpressure of 300 mbar, followed by closing the gases and monitoring the pressure loss over 2 min. If the loss was below 30 mbar min^−1^, the cell sealing was considered acceptable. The cell temperature was then raised to 120 °C under nitrogen flow (100 mL min^−1^), with the heating jackets for the cathode and anode gas supply set to 130 °C. After reaching 120 °C, the gases were switched to dry hydrogen and oxygen at a stoichiometry *λ*_anode/cathode_ of 1.5/45.5 (H_2_/O_2_), which means under application of 0.1 A cm^−2^, a H_2_ flow rate of 50 mL min^−1^ and O_2_ flow rate of 166 mL min^−1^ for all GDEs. At this point, the current density was increased to 100 mA cm^−2^. The temperature was increased to 160 °C, and the MEA characterization protocol started (**Figure** **8**). The initial characterization of BoT was applied according to Figure 8. An external potentiostat Modulab2100A (Amite, Solation Analytical) equipped with an external booster (12 V 20 A^−1^) was connected to the cell for the EIS, cyclic voltammetry, and linear sweep voltammetry measurements. The minimum voltage for the MEA testing was set to 100 mV so that the turning point of the polarization curve (galvanostatic potential‐current (UI)) was determined by this value, as well as for the EIS measurements. After the BoT protocol, the constant load test was continued for at least 100 h. Afterward, the final characterization EoT was performed analog to the BoT protocol (Figure 8). At least two MEAs per GDE modification were tested.

##### DRT Analysis

The electrochemical impedance data were analyzed using DRT using Equation (1).^[^ ^31^ ^]^

(1) $$Z\left(f\right)=\;R_{\infty }+R_{\text{pol}}\int_{0}^{\infty }\frac{g\left(\tau \right)}{1+2i\pi f\tau }d\tau$$

Where $R_{\infty }$ is the ohmic resistance, $R_{\text{pol}}$ is the polarization resistance, *g* is the DRT function, *τ* is the relaxation time, and *f* is the frequency. DRT tools developed by the Ciucci group were used to calculate the DRT function with a regularization parameter of 10^−3^, chosen from an initial assessment to find a trade‐off between low residuals, selectivity, and minimal oscillations for PEMFCs. The Gaussian method of discretization was utilized with a 1st order of regularization derivative. Using the SciPy library of Python and Akaike information criterion function from DRT tools, impedances associated with different electrochemical processes in the DRT data were extracted. The peak separation and evaluation were carried out by identifying an initial set of peaks, and a vector data set was created based on these initial peaks. Then, prioritizing the higher peak heights, a Gaussian fitting was performed, and then, using the difference between the fitted data and the previous data, an iterative loop was created to minimize the difference to 10^−6^. The area under the curve was computed by an integration function and validated with a composite trapezoidal rule. The integrated peak area represents the resistance and the center of the maximum of the peak the rate of the process associated with the resistance.

##### Cyclic Voltammetry Peak Deconvolution

Peak deconvolution and computations of the area under the peaks for cyclic voltammetry measurements were carried out utilizing MATLAB. Herein, we used Gaussian–Lorentzian cross‐product function to fit peaks based on constraints in potential values (in *x*‐axis).

(2) $$y=y_{0}+\frac{A}{1+e^{0.5\left(1-s\right){\left(\frac{x-x_{c}}{w}\right)}^{2}}s{\left(\frac{x-x_{c}}{w}\right)}^{2}}$$

Where, *y* and *x* in Equation (2) are the respective axes for current and potential, *y*_0_ is the baseline, *x*_c_ is the center of the peak, *A* is the amplitude or height of the peak, *w* is the width of the peak at the half maximum peak height, and *s* is the shape factor that defines the ratio between Gaussian and Lorentzian components (*s *= 0 for pure Gaussian and *s *= 1 for pure Lorentzian). The constraint for quinone/hydroquinone redox peak was between 0.4 and 0.6 V, and for the Fe^2+^/^3+^ redox peak, it was between 0.7 and 0.9 V. The *y*_0_ baseline was fitted using eight or more anchor points using an iteration method minimizing the residuals, allowing the baseline to have a linear function, a piecewise polynomial function (spline), or a basis spline. Two peaks using the Gaussian–Lorentzian cross‐product were used to fit the cumulative peak, which also used the iteration method, minimizing residual values.

## Conflict of Interest

The authors declare no conflict of interest.

## Supporting information

Supplementary Material

## Data Availability

The data that support the findings of this study are available from the corresponding author upon reasonable request.
