# Supplementary material for: Influence of Ink Composition and Drying Technique on the Performance and Stability of Fe–N–C‐Based High‐Temperature Proton Exchange Membrane Fuel Cells
Source: ChemSusChem. 2025 Jul 14;18(16):e202500905. doi: 10.1002/cssc.202500905 (PMC12330310; doi:10.1002/cssc.202500905)
Supplement: Supplementary file 1 — Supplementary Material [file CSSC-18-e202500905-s001.pdf]

Supporting Information

Influence of Ink Composition and Drying Technique on the Performance and Stability of Fe-N-C-based High-Temperature Proton Exchange Membrane Fuel Cells

Tanja Zierdt*[a, b], Md Raziun Bin Mamtaz[c], Tom Eek[a, d], Julia Müller-Hülstede[a], Steffen Rehse[a], Quentin Meyer[c], Dana Schonvogel[a], Peter Wagner[a], Chuan Zhao[c], Michael Wark[d], K. Andreas Friedrich[b, e]

[a] T. Zierdt*, T. Eek, Dr. J. Müller-Hülstede, S. Rehse, Dr. D. Schonvogel, P. Wagner
Institute of Engineering Thermodynamics
German Aerospace Center (DLR)
Carl-von-Ossietzky-Str. 15, 26129 Oldenburg (Germany)

[b] T. Zierdt*, Prof. Dr. K. A. Friedrich
Institute for Building Energetics, Thermotechnology and Energy Storage (IGTE)
University of Stuttgart
Pfaffenwaldring 31, 70569 Stuttgart (Germany)

[c] Md R. B. Mamtaz, Dr. Q. Meyer, Prof. Dr. C. Zhao,
School of Chemistry
The University of New South Wales
NSW, 2052, Sydney (Australia)

[d] T. Eek, Prof. Dr. M. Wark
Institute of Chemistry
Carl von Ossietzky University Oldenburg
Carl-von-Ossietzky-Str. 9-11, 26129 Oldenburg (Germany)

[e] Prof. Dr. K. A. Friedrich
Institute of Engineering Thermodynamics
German Aerospace Center (DLR)
Pfaffenwaldring 38-40, 70569 Stuttgart (Germany)

*E-mail: tanja.zierdt@dlr.de

Preliminary tests and variation of solvents and the freezing parameters

Impact of temperature

A GDE with same t-BuOH ink composition was prepared, but frozen at -80 °C instead of -26 °C. However, the CL surface was visibly very inhomogeneous, likely due to solvent/water crystal formation on top of the CL surface. The whole CL surface is sprinkled with spots and irregularities (Figure S 1-A). This approach was therefore not investigated further.

Impact of solvent

Cyclohexanol, ethylene glycol, acetonitrile, dimethyl sulfoxide as solvents have on the first glance attractive properties regarding factors like the solubility in water, possible miscibility gaps and viscosity. However, the system of water, PTFE, catalyst and the solvent is such complex that testing is needed, regardless the material characteristics. We found that **acetonitrile, dimethyl sulfoxide** did not homogenize or suffered from phase separation or severe coagulation after mixing and ultrasonication bath treatment for 15 min. with the PTFE solution (without catalyst). Quality of suspension of the solvent with the binder is a one of the main criteria for the suspension, so that acetonitrile and dimethyl sulfoxide were excluded for further tests due to inferior mixing quality. In comparison, the tests with **IPA** and **t-BuOH** had higher degree of homogenization, no flocculation and showed similar behavior. The **cyclohexanol** suspension also showed stable behavior.

For GDE fabrication cyclohexanol (Sigma Aldrich) was investigated, adapted from the freeze-drying of LT-Pt/C-GDEs Talukar et al.[1]. The suspension was prepared similar to the t-BuOH suspension. The GDE was placed into the freezer with -26 °C, however the CL did not solidify, which made this approach not applicable. A next test at -80°C led to solid CL. However, after the sublimating step, almost no adhesion to the GDL/MPL was visible and the detached CL exhibited “air pockets” at the interface (Figure S 1-B). This is assumed to stem from significant higher retention of the cyclohexanol from the GDL/MPL compared to t-BuOH and IPA solvents, which was visible by rough estimation of the contact angle via dropping the solvents and the PTFE/solvent mixture onto the GDL/MPL surface. The ink used by Talukdar[1] with cyclohexanol (and Nafion™) to fabricate a Pt-based LT-GDE via freeze drying cannot be transferred to HT-GDEs with Fe-N-C and PTFE binder. The goal is to generate a smooth, homogenous CL surface was not achieved with -80 °C or cyclohexanol with this specific catalyst material and ink composition. However, for other systems it might be considerable.

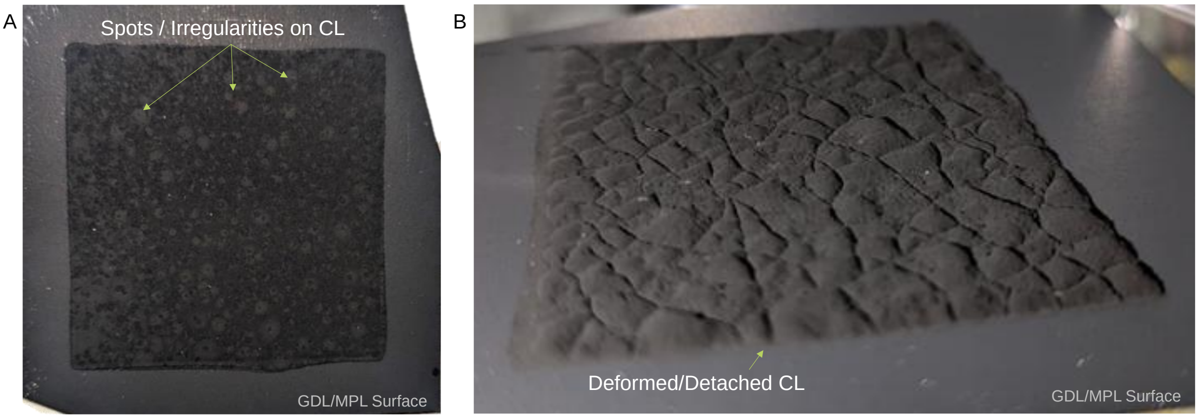

Figure S 1. A) GDE after freezing at -80°C and subsequent sublimation, made from t-BuOH suspension. B) GDE after freezing at -80°C sublimation, made from cyclohexanol suspension.

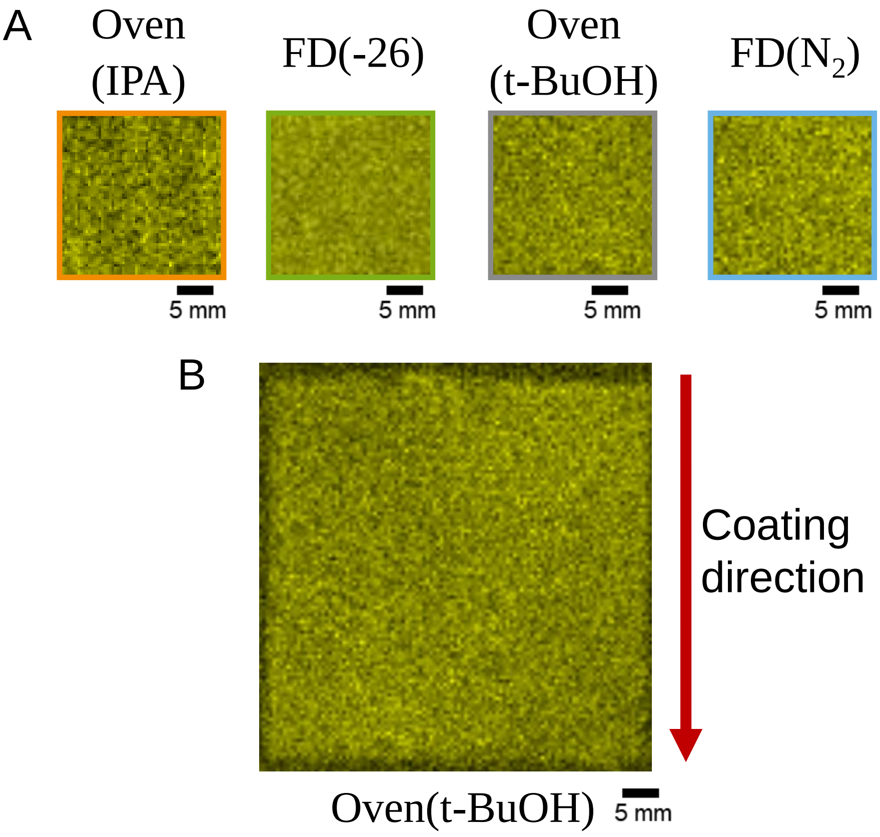

Figure S 2. A) Iron mapping from µ-XRF of the GDEs (2.3 x 2.3 cm pieces) for MEA single cell testing. B) Exemplarily for all samples the whole GDE (5 x 5cm) iron mapping of the Oven(t-BuOH) GDE. The doctor blade coating direction is from top (of the shown image) to bottom.

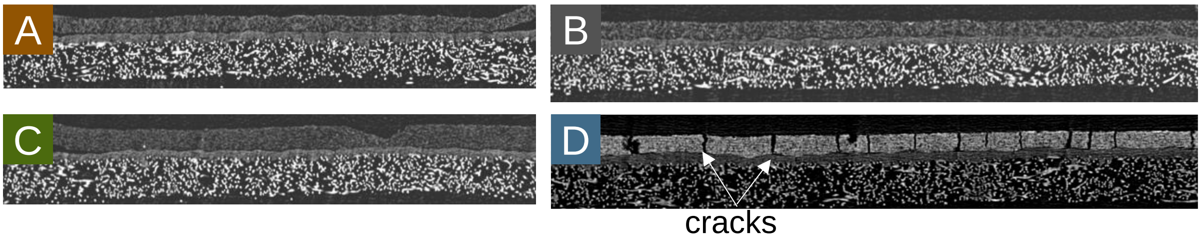

Figure S 3. A-D) 2-D µ-CT images of the GDEs A) Oven(IPA) B) Oven(t-BuOH) C) FD(-26) and D) FD(N_2).

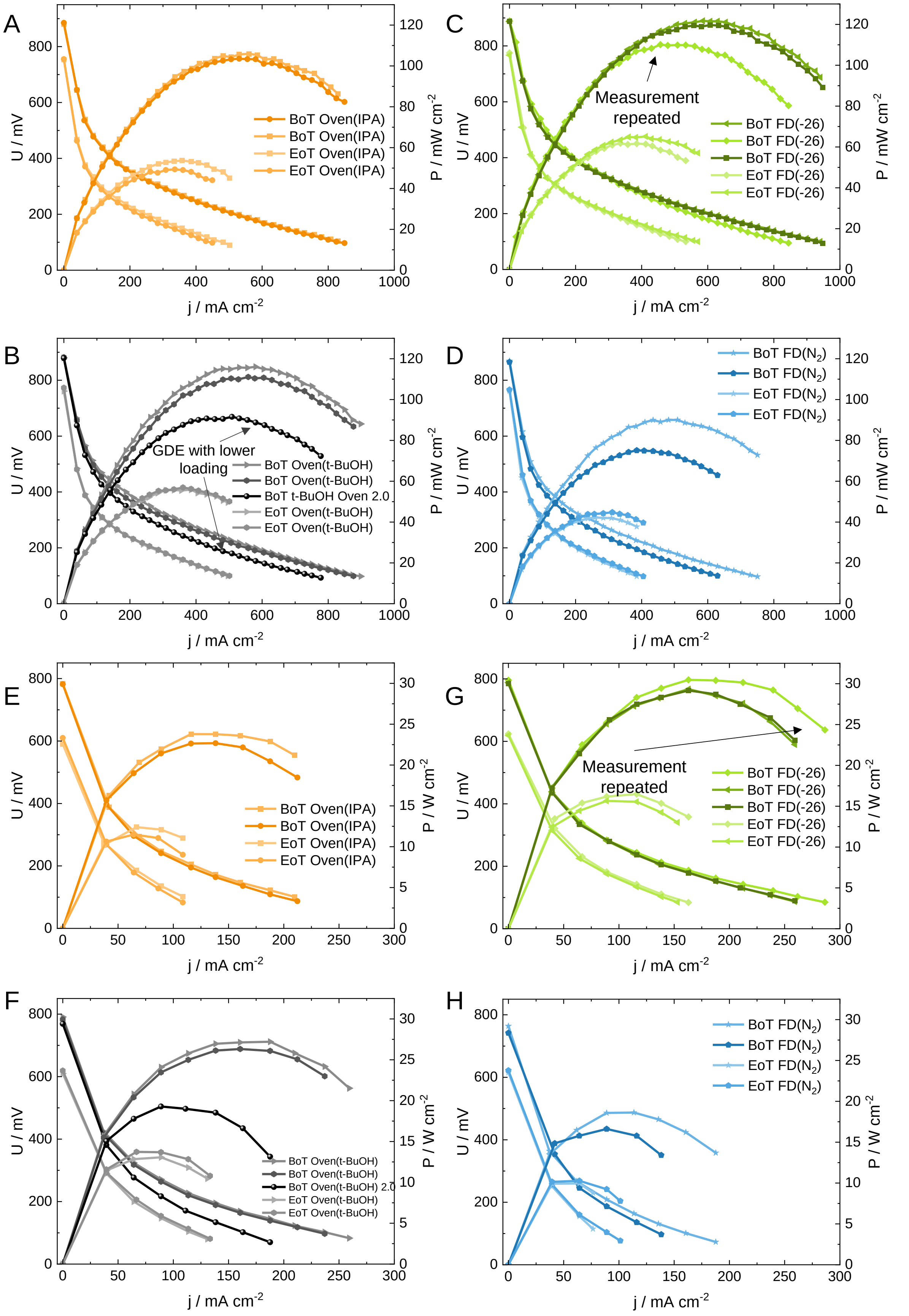

Figure S 4. A-D) Polarization curves, recorded with a step size of 0.1 A, of HT-PEMFC measurement with a cell compression of 0.75 MPa (477 mbar) at T=160 °C with ʎ 1.5/45.5 H_2/O_2 operation at BoT (in bold color) and EoT after > 100 h (in lighter color). E-H) Polarization curves with ʎ 1.5/2.0 H_2/air operation. Celtec®-based Pt/C anode (Celtec High Temperature PEM Fuel Cell, P1100W, Fuel Cell Store) served as anode. The Fe-N-C cathode GDE are named in the figure.

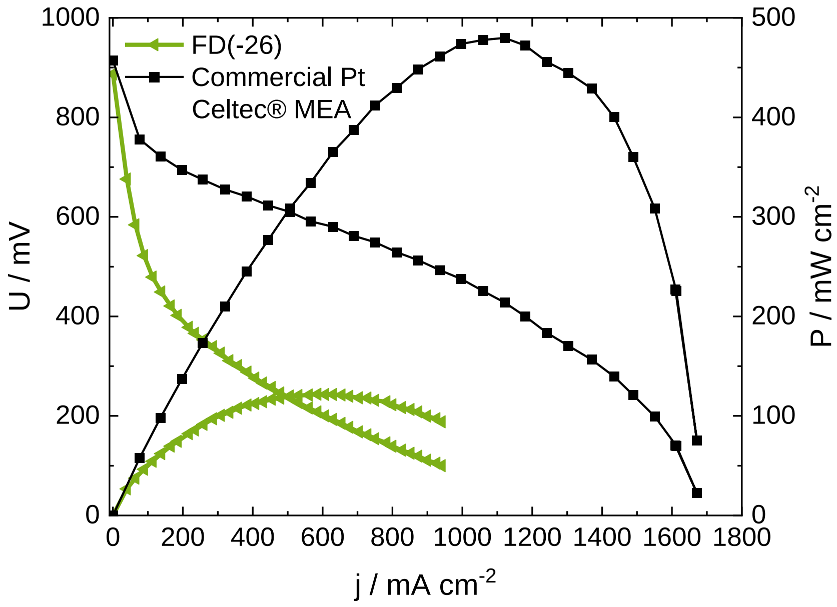

Figure S 5. Comparison of Fe-N-C MEA to Pt-based commercial MEA (Pt-alloy cathode, Pt-anode, Celtec® PBI membrane). HTPEMFC test at 160 °C with H_2/O_2 supply.

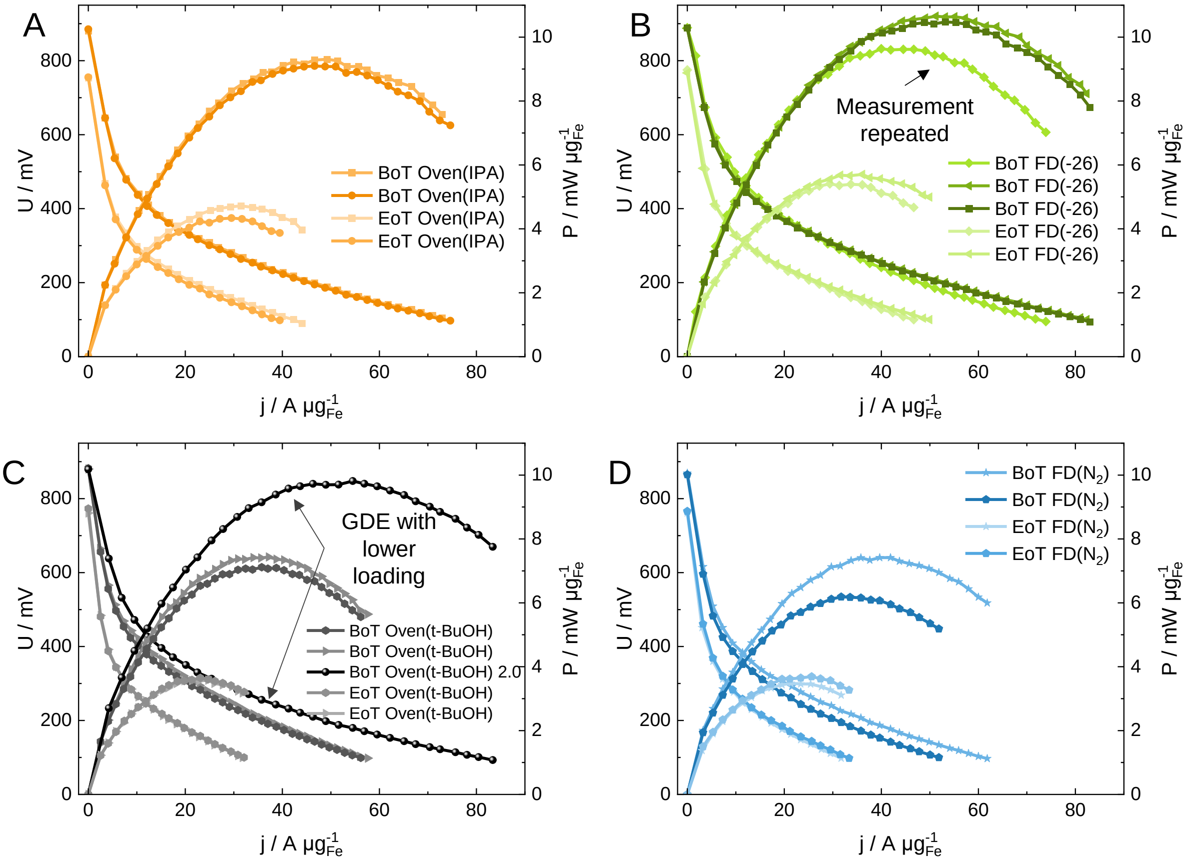

Figure S 6. A-D) Mass-normalized depiction of the polarization curves, recorded with a step size of 0.1 A, of HT-PEMFC measurement with a cell compression of 0.75 MPa (477 mbar) at T=160 °C with ʎ 1.5/45.5 H_2/O_2 operation at BoT (in bold color) and EoT after > 100 h (in lighter color). Celtec®-based Pt/C anode (Celtec High Temperature PEM Fuel Cell, P1100W, Fuel Cell Store) served as anode. The Fe-N-C cathode GDE are named in the figure.

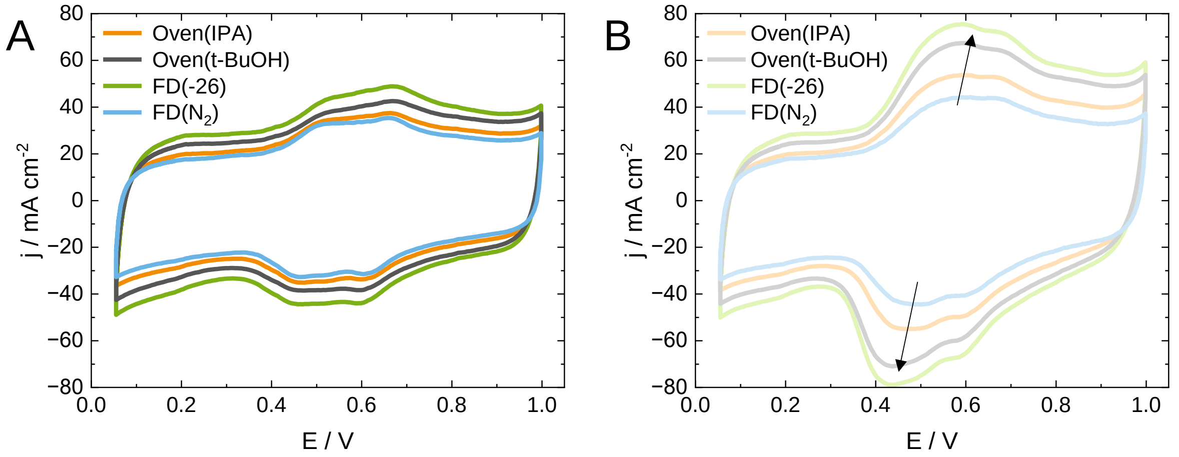

Figure S 7. A) Cyclic voltammograms of cathode at BoT and B) EoT characterization from two measurements. Recorded with 100 mL min^{-1} H_2 (anode) / N_2 (cathode) operation. HT-PEMFC measurement with a cell compression of 0.75 MPa (477 mbar) at T=160 °C.

Table S 1 Power density data from the polarization curves at BoT and EoT during H_2/O_2 and H_2/air operation. If no EoT characterization was performed the values are marked with an "x".

MEA	**Mass specific peak power density**					**Area specific peak power density**			
	/ mW µg$_{Fe}^{-1}$ H_2/O_2		/ mW mg$_{Fe\text{-}N\text{-}C}^{-1}$ H_2/O_2	/ mW µg$_{Fe}^{-1}$ H_2/air		/ mW cm^{-2} H_2/O_2		/ mW cm^{-2} H_2/air	
	BoT	*EoT*	*BoT*	*BoT*	*EoT*	*BoT*	*EoT*	*BoT*	*EoT*
Oven (IPA)	9.3	4.7		2.1	1.1	106	54	24	12
	9.1	4.3	41.4	2.0	1.0	104	49	23	11
FD(-26)	9.6	5.4		2.6	1.4	110	62	30	16
	10.5	x		2.6	x	120	x	29	x
	10.7	5.7	45.1	2.6	1.4	122	65	29	16
Oven (t-BuOH)	7.4	3.6		1.74	0.8	116	56	27	13
	7.1	3.6		1.69	0.9	111	57	26	14
Oven (t-BuOH) 2.0	9.8	x		2.1	x	92	x	19	x
FD(N_2)	7.4	3.5	33.3	1.5	0.8	90	42	19	10
	6.2	3.7		1.4	0.8	75	45	17	10

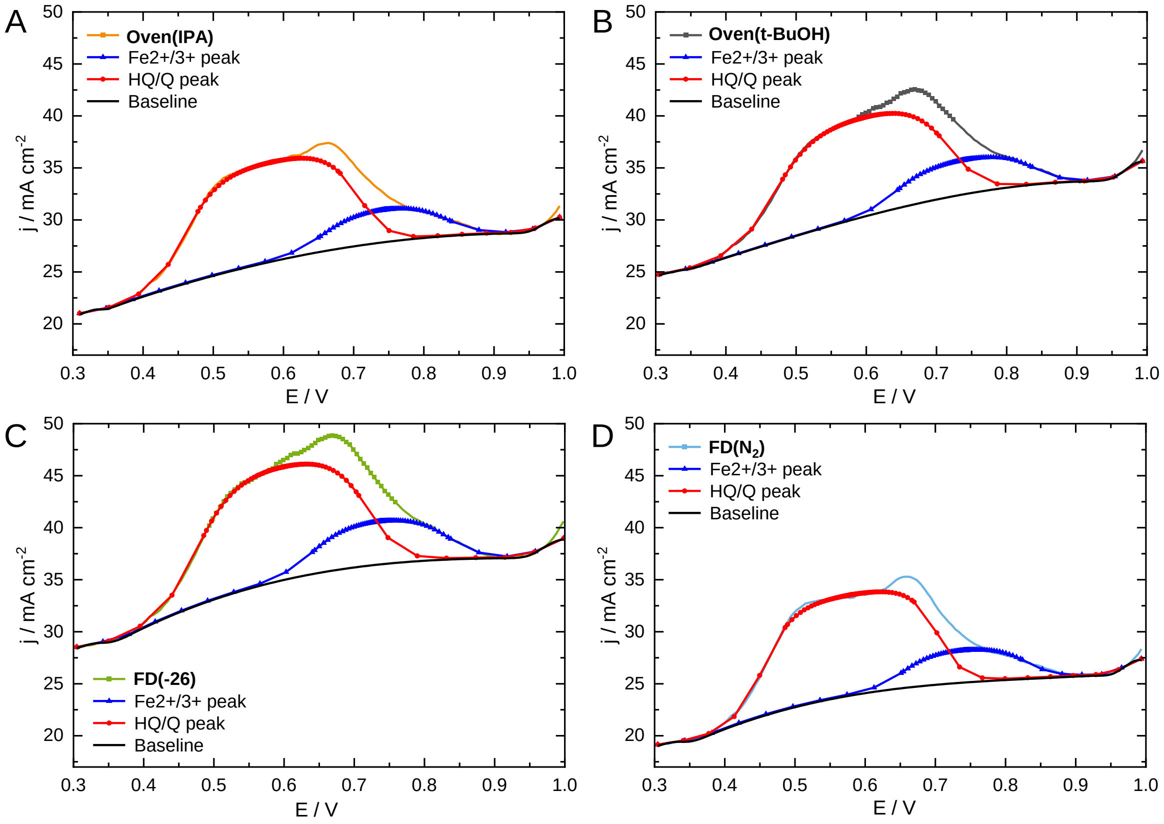

Figure S 8. Results from the Gaussian Lorentzian cross product function fitting of the CVs at BoT. The HQ/Q redox peak is fitted between 0.4 and 0.6 V and for the $Fe^{2+}/^{3+}$ redox peak between 0.7 and 0.9 V. The baseline and the fittings of the two peaks are depicted together with the initial CV data.

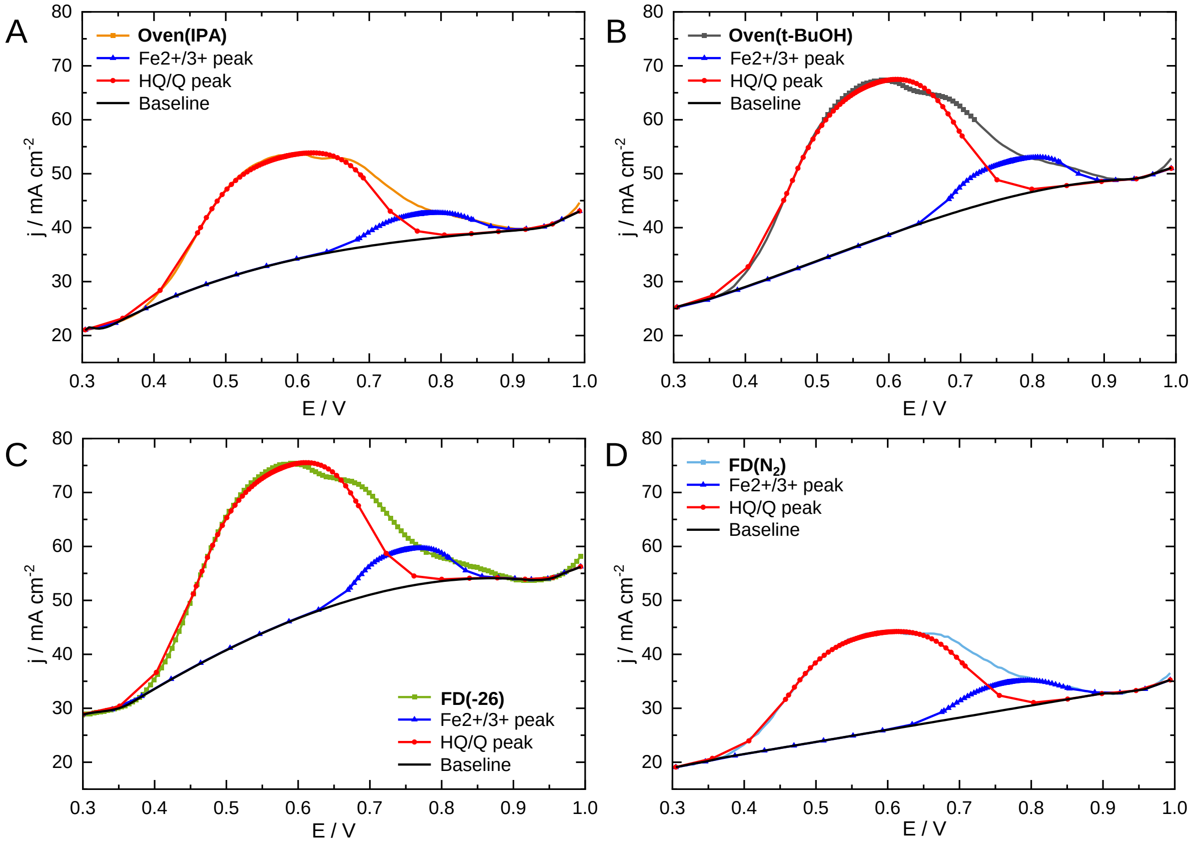

Figure S 9. Results from the Gaussian Lorentzian cross product function fitting of the CVs at EoT. The HQ/Q redox peak is fitted between 0.4 and 0.6 V and for the $Fe^{2+}/^{3+}$ redox peak between 0.7 and 0.9 V. The baseline and the fittings of the two peaks are depicted together with the initial CV data.

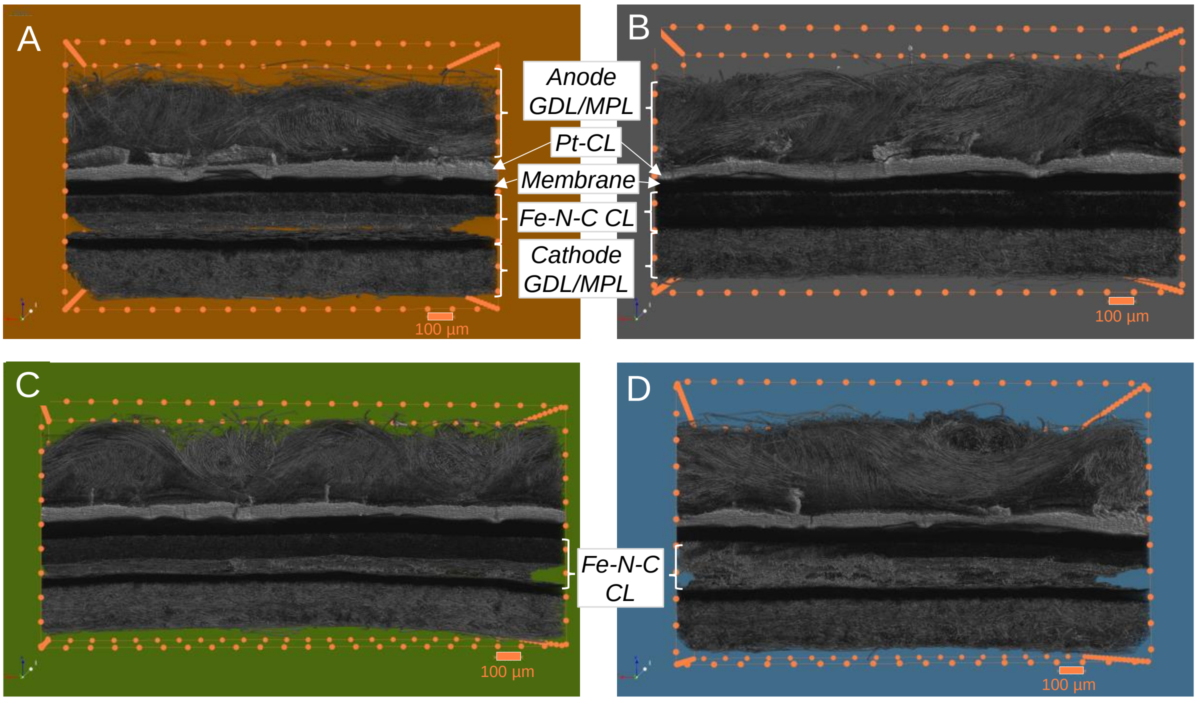

Figure S 10. 3-D µ-CT images of the MEAs after more than 100 h of operation. A) Oven(IPA) MEA B) Oven(t-BuOH) MEA C) FD(-26) MEA D) FD(N_2) MEA.

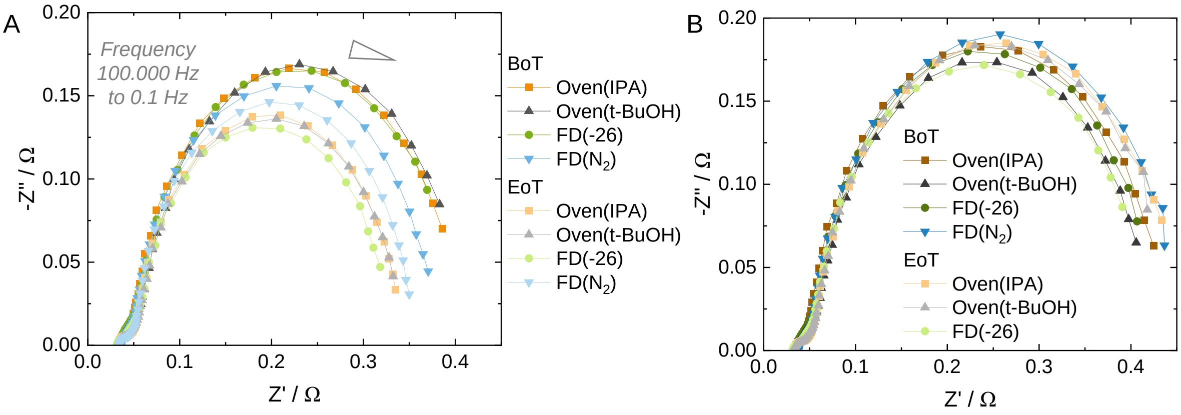

Figure S 11. A) Nyquist plot from BoT and after 100 h at EoT during H_2/O_2 operation and B) during H_2/air operation at 100 mA cm^{-2}.

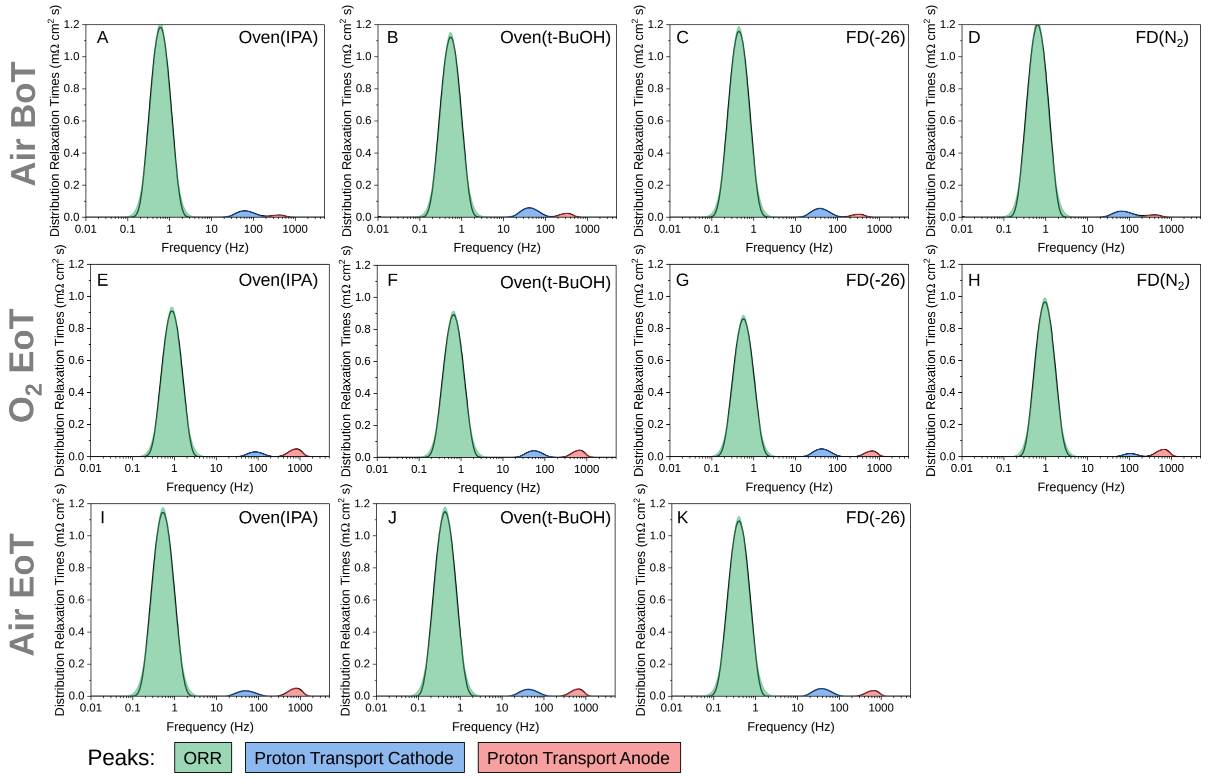

Figure S 12. DRT plots for all MEAs at BoT H_2/air supply and EoT H_2/air and H_2/O_2 supply at 100 mA cm^{-2}. The first peak belongs to the ORR-, the second peak to proton transport cathode- and the third to proton transport anode resistance.

Table S 2. Resistance values and rates from the DRT analysis.

MEA name		ORR resistance / mΩ cm^2	Cathode proton transport resistance / mΩ cm^2	Anode proton transport resistance / mΩ cm^2	ORR rate / s^{-1}	Cathode proton transport rate / s^{-1}	Anode proton transport rate / s^{-1}
Oven(IPA)	O_2 BoT	1481	51	12	0.48	47	336
Oven(t-BuOH)		1481	51	12	0.39	29	245
FD(-26)		1477	67	15	0.34	27	245
FD(N_2)		1367	51	---	0.74	79	---
Oven(IPA)	Air BoT	1619	49	13	0.60	64	413
Oven(t-BuOH)		1528	70	20	0.54	43	316
FD(-26)		1534	57	30	0.44	39	306
FD(N_2)		1642	51	38	0.69	68	413
Oven(IPA)	O_2 EoT	1202	30	43	0.86	86	780
Oven(t-BuOH)		1196	44	37	0.66	54	632
FD(-26)		1151	55	30	0.57	42	632
FD(N_2)		1267	18	39	0.96	105	632
Oven(IPA)	Air EoT	1658	41	45	0.52	50	758
Oven(t-BuOH)		1642	51	38	0.43	43	632
FD(-26)		1121	49	30	0.40	38	612
FD(N_2)		1642	51	38	---	---	---

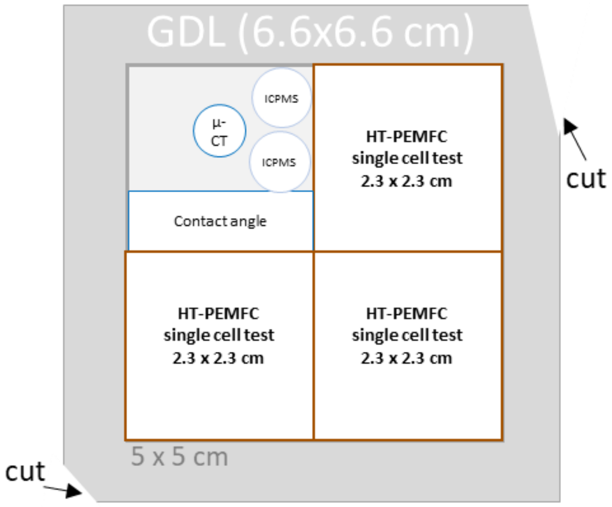

Figure S 13. Schematic representation of the GDE and representation how the pieces were used for physical and electrochemical analysis. The CL suspension was applied from top to bottom by doctor blade coating. The cuts were used to track if there is an effect from the coating direction.

References

[1] K. Talukdar, S. Delgado, T. Lagarteira, P. Gazdzicki, K. A. Friedrich, *J Power Sources* **2019**, *427*, 309.
